# The Molecular Links between Cell Death and Inflammasome

**DOI:** 10.3390/cells8091057

**Published:** 2019-09-10

**Authors:** Kwang-Ho Lee, Tae-Bong Kang

**Affiliations:** 1 Department of Biotechnology, College of Biomedical & Health Science, Konkuk University, Chungju 27478, Korea; 2 Research Institute of Inflammatory Diseases, Konkuk University, Chungju 27478, Korea

**Keywords:** inflammasome, apoptosis, necroptosis, programmed cell death, Caspase-8, RIPK1/3, MLKL, PGAM5, DRP1

## Abstract

Programmed cell death pathways and inflammasome activation pathways can be genetically and functionally separated. Inflammasomes are specialized protein complexes that process pro-inflammatory cytokines, interleukin-1β (IL-1β), and IL-18 to bioactive forms for protection from a wide range of pathogens, as well as environmental and host-derived danger molecules. Programmed cell death has been extensively studied, and its role in the development, homeostasis, and control of infection and danger is widely appreciated. Apoptosis and the recently recognized necroptosis are the best-characterized forms of programmed death, and the interplay between them through death receptor signaling is also being studied. Moreover, growing evidence suggests that many of the signaling molecules known to regulate programmed cell death can also modulate inflammasome activation in a cell-intrinsic manner. Therefore, in this review, we will discuss the current knowledge concerning the role of the signaling molecules originally associated with programmed cell death in the activation of inflammasome and IL-1β processing.

## 1. Introduction

Homeostasis is a principle property of living organisms and it is maintained at the systemic, tissue, and cellular levels through the homeostatic control system. To maintain homeostasis, cells use specialized sensors to monitor internal and external changes. The immune system is composed of specialized cells that sense infection or tissue damage, and the stimulation of the immune sensors can lead to inflammation or cell death induction. Although both inflammation and cell death are protective responses, the extent of their mechanistic overlap is not well defined. The new data emerging from recent studies, however, are beginning to shed light on this important issue.

In this review, we discuss the recent advances in our knowledge and understanding of the role of the protein regulators of programmed cell death in inflammasome activation and interleukin-1β (IL-1β) processing.

## 2. Inflammasome

The inflammasome is a macromolecular signaling complex composed of sensors that recognize microbial components and cell injury, an adaptor protein apoptosis-associated speck-like protein containing a caspase recruitment domain (ASC), and caspase-1. The formation of such a complex leads to the activation of caspase-1, which induces the cleavage and secretion of pro-inflammatory cytokine interleukin-1β (IL-1β) and IL-18 [1]. 

Different types of inflammasome sensors are activated in response to different stimuli [2]. They are categorized according to their structural characteristics into nucleotide-binding domain–like receptors (NLRs), and absent in melanoma 2 (AIM2)-like receptors (ALRs) [2]. 

The NLR family pyrin domain containing 1 (NLRP1) inflammasome is activated by the *Bacillus anthracis* lethal toxin, *Toxoplasma gondii*, and host intracellular ATP depletion [3,4,5,6,7]. In addition, the infection of the lymphocytic choriomeningitis virus (LCMV) triggers NLRP1 inflammasome activation in hematopoietic progenitor cells [8].

The NLRP3 inflammasome responds to damage- or danger-associated molecular patterns (DAMPs), such as ATP, bacterial pore-forming toxins, viral RNA, and particulate matters [3,9,10,11], and it is also activated by some bacteria [12,13,14] and viruses, including the influenza virus, encephalomyocarditis virus (EMCV), and Sendai virus [15,16,17,18,19]. 

The NLR family caspase activation and recruitment domain (CARD)—containing protein 4 (NLRC4) inflammasome—is activated in response to bacterial flagellin [20], or to proteins from the bacterial type III secretion system [21,22,23,24,25,26]. 

Interferon-inducible AIM2 contains a pyrin domain (PYD) and HIN-200 domains and induces inflammasome assembly in response to the cytosolic dsDNA of cytosolic bacteria [27,28,29,30,31], and to DNA viruses such as vaccinia virus (VACA) and mouse cytomegalovirus (MCMV) [28,32].

Therefore, inflammasome is an innate immune system that responds to a broad range of self- or foreign-insults, including bacteria and viruses. However, it should be noted that several bacteria and viruses have developed evolutionary strategies in order to evade inflammasome activation. 

Some bacteria, such as *Pseudomonas aeruginosa* and *Yersinia Species*, utilize the effector molecules that are secreted by bacteria into the cells of their host in order to suppress inflammasome activation. For example, ExoU and ExoS from *P. aeruginosa*, and YopE, YopM, and YopT from *Yersinia spp.* inhibit caspase-1 activation and the subsequent IL-1β processing [33,34,35].

Poxviruses produce PYD-only proteins (POPs), and serine proteinase inhibitor (serpin) homologs, such as CrmA, SP1/2 (SPI1/2), and Serp2, were shown to abolish the activity of caspase-1, leading to the inhibition of IL-1β processing, and several viral proteins—influenza virus protein NS1, vaccinia virus protein B15R, and poxvirus proteins MC53L and MC54L—also inhibit caspase-1 activation [33,34,35].

Of all of the inflammasomes, NLRP3 is the best-characterized and most extensively studied inflammasome because of its role in many infectious and inflammatory diseases [36,37]. Moreover, the interplay of NLRP3 inflammasome with cell death systems is relatively well characterized compared with other inflammasomes [38]. 

### 2.1. Canonical Inflammasome Activation

**Figure 1:**
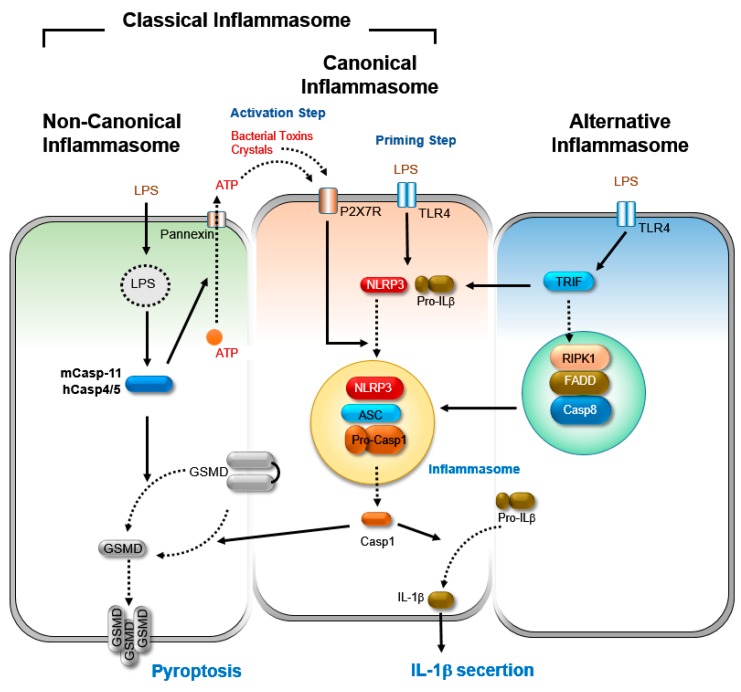
A simple depiction of signaling pathways for the activation of classical NLRP3 inflammasome (canonical and non-canonical) and alternative inflammasome. Canonical inflammasome activation includes a priming and activation step. The priming step, by pathogen-associated molecular patterns (PAMPs) and cytokines, leads to the expression of inflammasome components, NLRP3, and pro-IL-1β through the NF-κB pathway. The activation step is triggered by damage-associated molecular patterns (DAMPs), such as ATP, crystals, pore-forming toxins, and metabolites, which induce the formation of the NLRP3 inflammasome complex, where caspase-1 is activated. Activated caspase-1 in turn cleaves pro-IL-1β for the production and secretion of active IL-1β. It also cleaves gasdermin D (GSMD), which leads to the formation of membrane pores and triggers pyroptosis. Non-canonical NLRP3-inflammasome is initiated by sensing cytosolic LPS by caspase-11/-4/-5. Then, activated caspase-11/-5/-4 cleaves GSDMD, which leads to pyroptosis. In parallel, the activated caspase-11/-4/-5 activates pannexin-1, leading to ATP release and K^+^ efflux in order to derive NLRP3 inflammasome activation and IL-1β secretion. In contrast to classical inflammasome activation, alternative inflammasome activation can be triggered by a single signal. LPS stimulation induces the activation of NLRP3 inflammasome via a TLR4-TRIF-RIPK1-FADD-CASP8 signaling axis, independently of the potassium efflux. The alternative inflammasome activation does not induce pyroptosis.

It is generally accepted that the NLRP3 inflammasome is activated in a two-step process: the priming step and the activation step (Figure 1). The primary macrophages, derived from mouse bone marrow, show undetectable or minimal levels of NLRP3 inflammasome activation upon stimulation with the NLRP3 stimuli. However, the pretreatment of cells with pathogen-associated molecular patterns (PAMPs), such as LPS, referred to as the priming step, induces the expression of NLRP3 and pro-IL-1β through the activation of NF-κB-dependent, so as to produce a sufficient amount of the proteins needed for optimal NLRP3 inflammasome activation [39]. 

In addition to this transcriptional regulation, recent studies have implicated that the priming step also contributes to NLRP3 inflammasome activation at post-transcriptional levels. The deubiquitinating enzyme, BRCC3, promotes the deubiquitination of the NLRP3 at the priming step, which is crucial for NLRP3 inflammasome activation [40,41]. It has also been reported that the priming step is required for the c-Jun N-terminal kinase (JNK1)-mediated phosphorylation of NLRP3, which is essential for NLRP3 deubiquitination [42]. Moreover, the phosphorylation of ASC by IKKi/IKKε, which facilitates the peri-nuclear translocation of ASC from the nucleus, also occurs during the priming step [43].

The activation step of NLRP3 inflammasome is triggered by various pathogen-associated molecular patterns (PAMPs) and danger-associated molecular patterns (DAMPs), such as extracellular ATP, pore-forming toxins, and particulate matters [2,11]. These stimuli promote the assembly of the NLRP3 inflammasome complex, the activation of caspase-1, and subsequently the maturation and secretion of IL-1β and IL-18. Several cellular events, such as mitochondrial dysfunction, ROS production, potassium efflux, and cell swelling, have been proposed as upstream signals for NLRP3 inflammasome activation [2,11]. Among them, the reduction of intracellular concentrations of potassium ions (K^+^) has been reported to be a common feature after interaction with most of the stimuli [10,44]. Therefore, the efflux of intracellular K^+^ is proposed to be the common upstream link among the various activators of NLRP3 inflammasome [10]. However, the downstream molecular mechanism for NLRP3 activation in response to potassium efflux has remained unclear. A mitotic serine/threonine kinase NEK7 has recently been identified as a critical component of NLRP3 inflammasome activation. NEK7 has been shown to promote the assembly and activation of the NLRP3 inflammasome via direct interaction with the NLRP3 [26,45,46,47].

In addition to the processing of pro-inflammatory interleukins, inflammasome activation also leads to a necrosis-like programmed cell death called pyroptosis. Pyroptosis is initiated by the cleavage of gasdermin D (GSDMD) by activated caspase-1 in inflammasome [48,49,50]. Then, the N-terminal fragment of the GSDMD subsequently associates with the plasma membrane and executes lytic cell death by forming the pores in the membrane [48,49,50]. 

### 2.2. Non-Canonical Inflammasome Activation

The cleavage of GSDMD and the subsequent pyroptosis also occurs in another cellular context. Recently, a new pathway of inflammasome activation, called non-canonical NLRP3 inflammasome activation, has been revealed, which is critical for defense against intracellular Gram-negative bacteria, but not Gram-positive ones [2,51,52]. Although the non-canonical NLRP3 inflammasome activation also leads to IL-1β secretion and pyroptotic cell death, its mechanisms differ from the canonical pathway, which requires the two step-process. 

Unlike canonical NLRP3 inflammasome activation, non-canonical activation requires upstream caspases, such as caspase-4/-5 in humans and caspase-11 in mice, as a receptor, and its activation is initiated through the direct recognition of intracellular LPS or Lipid-A by the caspase-4/-5/-11 [51,53,54]. The binding of ligands leads to the activation of caspase-4/5/11, followed by the cleavage of the pore-forming protein GSDMD. The formation of membrane pores by cleaved GSDMD facilitates pyroptosis, potassium efflux, and subsequent NLRP3 inflammasome activation. 

Notably, there are subtle differences in the activation mode between murine and human cells. In murine macrophages, the priming step is crucial for non-canonical inflammasome activation, because of the low expression level of caspase-11 in the resting cells. However, diverse human cells, including monocytes, keratinocytes, and epithelial cells, express a high-level of caspase-4. Therefore, in human cells, the priming step is not required for LPS-induced non-canonical inflammasome activation [53]. Similar functional differences between humans and mice have been shown in the recognition of *Francisella spp.*, which proliferates in the host cell cytosol. AIM2 inflammasome has been shown to be critical in the murine response to this bacteria, however, the human monocyte utilizes sensors other than AIM2 for *Francisella* detection [55,56], which indicates that there are some evolutionary changes in pathogen sensing between humans and mice. 

### 2.3. Alternative Inflammasome Activation

Although it has been well established that NLRP3 inflammasome is triggered by a two-step mechanism, compelling evidence has been presented that inflammasome activation induced by TLR4 signaling does not require a second co-stimuli. The pathway was therefore named alternative inflammasome activation. [57,58]. Although alternative inflammasome activation requires ASC, and yields caspase-1 activation and IL-1β secretion, it differs from classical inflammasome activation (canonical and non-canonical) in that it requires the adaptor protein TIR-domain-containing adapter-inducing interferon-β (TRIF) and caspase-8 activation. In addition, it proceeds independently of the potassium (K^+^) efflux, and is not accompanied by pyroptosis. Intriguingly, the alternative inflammasome activation seems to be species- and cell type-specific, because it occurs in human monocytes but not in murine cells [57]. 

## 3. Programmed Cell Death

Programmed cell death, or apoptosis, is an essential intracellular mechanism for maintaining homeostasis in multicellular organisms, and is widely used for removing unwanted cells, such as damaged cells [59,60]. However, apoptosis is no longer solely synonymous with programmed cell death, because of the identification of other forms of programmed death, such as necroptosis and pyroptosis. Each death depends on different mechanisms and yields different results in vivo [61]. Apoptosis is generally accepted as a programmed cell death machinery, which essentially does not elicit inflammation. However, necroptosis (as discussed below) and pyroptosis are inflammatory types of death that are characterized by cell swelling, membrane pore formation, and plasma membrane rupture [60,62]. Therefore, both necroptosis and pyroptosis result in the release of inflammatory intracellular contents, leading to inflammation. However, they have distinct functions and signaling pathways. While necroptosis is mostly observed as a back-up system that is initiated when apoptosis is blocked [63,64], pyroptosis is an inflammasome-mediated primary cellular response following the sensing of a broad range of PAMPs and DAMPs. Mixed lineage kinase domain-like protein (MLKL) and GSDMD act as executioners for necroptosis and pyroptosis, respectively. GSDMD is activated by caspase-1 or caspase-11, but MLKL is dependent on the kinase activity of RIPK3 [61].

### 3.1. Apoptosis

Apoptotic cell death has been divided into two broad categories: (1) the intrinsic pathway, which is activated by cellular stress and injury, and (2) the extrinsic pathway, which is initiated by the triggering of death receptors. Both pathways lead to the activation of effector caspases, such as caspase-3, -6, and -7, resulting in apoptosis.

#### 3.1.1. Intrinsic Apoptosis

**Figure 2:**
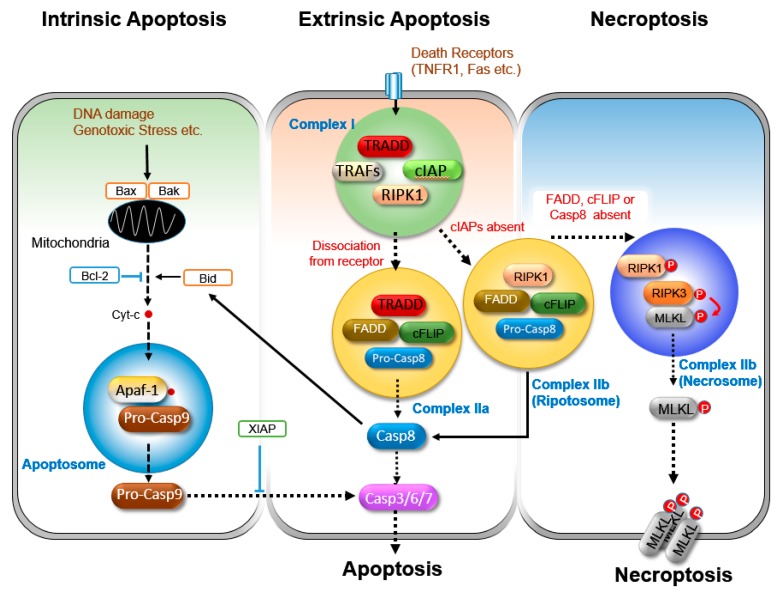
A simple depiction of the pathways for intrinsic and extrinsic apoptosis and necroptosis. The triggering of intrinsic apoptosis induces the mitochondrial membrane potential disruption and the release of cytochrome C into the cytoplasm. Then, cytochrome C interacts with apoptotic protease activating factor 1 (APAF1) and pro-caspase-9 to form a complex, named the apoptosome, where pro-caspase 9 is activated by the cleavage. The activated caspase-9 then activates the executioner caspases-3, -6, and -7 to execute cell death. This pathway is promoted by pro-apoptotic members of the Bcl2 family, such as BAK, BAX, and BID, activated by caspase-8, and is suppressed by anti-apoptotic proteins BCL-2, BCl-XL, and XIAP. The extrinsic apoptosis pathway is initiated by the activation of the death receptors of the TNF super family. Upon TNF stimulation, a receptor bound complex (complex I) is formed by RIPK1, TRADD, cIAP, and TRAFs, which essentially prevents the transition to the cell death pathway. However, TRADD/RIPK1 dissociation from the receptor, or TNF stimulation in the absence of cIAPs activity, causes the formation of different complexes, called complex IIa or complex IIb (ripoptosome), by interacting with FADD and caspase-8, where caspase-8 is activated. Then, active caspase-8 induces apoptosis through the activation of effector caspase-3/-6/-7, or by the cleavage of BID to promote cytochrome C release. TNF stimulation under the inhibition of both caspase-8 activity and cIAPs leads to the formation of another complex IIb (necrosome), where RIPK1, RIPK3, and MLKL are activated through phosphorylation. Then, activated MLKL forms an oligomer and translocates to the plasma membrane to execute necroptosis.

The intrinsic apoptosis pathway is activated by various exogenous and endogenous stimuli, such as DNA damage, radiation, oxidative stress, ischemia, and growth factor withdrawal (Figure 2) [65]. This pathway is controlled by the pro- and anti-apoptotic members of the BCL-2 family, and a subset of caspases [65,66]. 

In this pathway, the functional consequence of proapoptotic signaling is mitochondrial membrane potential disruption and the release of cytochrome C in the cytoplasm. Then, cytochrome C forms a complex, the apoptosome, with apoptotic protease activating factor 1 (APAF1) and pro-caspase 9, where pro-caspase 9 is cleaved and activated. The activated caspase-9 then activates the executioner caspases-3, -6, and -7, committing the cell to death [67,68]. Cell death can be inhibited by the X-linked inhibitor of apoptosis (XIAP) via the suppression of active caspase-9. Anti-apoptotic proteins, such as BCL-2 and BCL-XL, inhibit mitochondrial cytochrome C release, while pro-apoptotic proteins, such as BAX, BAK, and BID, trigger mitochondrial cytochrome C release [69,70].

#### 3.1.2. Extrinsic Apoptosis

Extrinsic apoptosis is initiated by the death receptors of the tumor necrosis factor (TNF) superfamily, including Fas, TNF receptor I (TNFR-I), and the TNF-related apoptosis-inducing ligand (TRAIL) receptors, DR4 and DR5 [71,72,73]. Of these, the TNF-induced apoptosis pathway is the best characterized.

Upon TNF stimulation, the protein kinase RIPK1 and TNF receptor-associated DD (TRADD) are recruited to the TNF receptor through the DD domain and form a receptor bound complex (complex I), which essentially prevents the transition to the cell death pathway [74,75]. Within this complex, RIPK1 is ubiquitinated by cIAPs, driving the activation of NFκB [76]. However, TNF receptor dissociation from TRADD/RIPK1 results in the formation of different complexes (complex IIa) by recruiting Fas-associated proteins via the death domain (FADD), and subsequently caspase-8 through the death effector domain (DED)-mediated homotypic interactions with FADD, which gives rise to RIPK1-kinase independent caspase-8 activation [77]. In addition, TNF stimulation in the absence or inhibition of cIAPs activity leads to the formation of a similar complex (complex IIb, also called ripoptosome) without TRADD, where caspase-8 is activated in an RIPK1-kinase dependent manner [78,79,80,81]. Then, the activated caspase-8 induces cell death by directly activating the effector caspases, such as caspase-3 and caspase-7, or by cleaving BID (BH3-interacting domain death agonist), resulting in mitochondrial dysfunction and the subsequent release of cytochrome C, and the activation of caspases-9 and the subsequent activation of effector caspases to execute cell death [82,83]. 

#### 3.1.3. Regulation of Extrinsic Apoptosis

Apart from signaling for apoptosis, TNF induces the expression of several anti-apoptotic proteins, such as the cellular FLICE-inhibitory protein (cFLIP) and the inhibitor of apoptosis (IAP) proteins, cellular IAP1 (cIAP1) and cIAP2. cFLIP suppresses the pro-apoptotic activity of caspase-8 by dimerization with caspase-8 through its DED, and cIAP proteins promote the NF-κB-dependent gene transcription by the ubiquitination of RIPK1, thereby inhibiting the RIPK1-mediated recruitment of FADD and caspase-8 [78,79,84,85]. In addition to cIAPs, several other enzymes can modulate RIPK1 uniquitination and thus regulate apoptosis. A20/TNFAIP3 is a multi-functional enzyme essential for maintaining a balanced NF-κB-response in the TNFR signaling pathway [86]. A20 has two domains, one responsible for K63 deubiquitination, another for the addition of K48-linked ubiquitin to RIPK1, marking it for degradation [86]. Thereby, A20 suppresses both TNFR-induced NF-kB activation and RIPK1-mediated apoptosis by promoting RIPK1 degradation [86,87,88].

Another deubiqutinase, CYLD, is recruited to the TNFR1-signaling complex, where it promotes the cell death pathway by removing the ubiquitin chains from RIPK1 and facilitating the formation of the complex II [89,90,91,92,93]. 

### 3.2. Necroptosis

Necroptosis is a recently discovered mode of programmed cell death, morphologically characterized by cell swelling and plasma membrane rupture. The notion that necrotic cell death is controlled by a defined program has increased interest to study this type of death in regards to development and inflammation [94]. Many death receptors, traditionally associated with apoptosis, can also trigger necroptosis when caspase-8 is inhibited, indicating that blocking apoptosis rewires cells to undergo a necrotic type of death instead [62,63]. 

This is physiologically important, because many viruses have evolved strategies to suppress apoptosis by encoding inhibitors of apoptosis [95]. For example, poxviruses encode homologues of human serpins that inhibit caspase-1 and caspase-8 [96,97]. Therefore, the inhibition of caspase-8 by poxvirus might provide the condition for necroptosis initiation. Indeed, infection with the vaccinia virus (VV), a poxvirus strain, sensitizes the cells to TNF- induced necroptosis [98], while the RIPK1 or RIPK3 deficient cells are resistant to the TNF-induced necroptosis [99]. Human herpes simplex virus I (HSV-1) and HSV-2 also encode RIP homotypic interaction motif (RHIM)-containing modulators, such as ICP6 and ICP10, respectively, and modulate necroptosis [100,101]. Interestingly, HSV-1 triggers necroptosis in mouse cells, while it inhibits necroptosis in human cells [102]. In addition to the DNA viruses, the RNA viruses also derive necroptosis. Sendai virus triggers necroptosis in the absence of caspase activity [103], and influenza A virus (IAV) elicits ZBP1-RIPK3 dependent necroptosis [104]. In addition, mice lacking necroptosis fail to control viral infections, including vaccinia virus, cytomegalovirus, and RNA virus [99,103,104,105,106,107].

Some viruses develop strategies to counteract or inhibit necroptosis, so as to escape from elimination. 

VV and MCMV encode RHIM-containing modulators, such as E3L and M45, which inhibit IFN-induced RIPK3/MLKL-dependent necroptosis through the inhibition of ZBP1 and RIPK3 [106,108,109,110,111], and Epstein–Barr virus (EBV) encodes the latent membrane protein 1 (LMP1), which inhibits TNF-induced necroptosis via the modulation of RIPK1/3 ubiquitination [112]. A class of viral FLIP proteins (vFLIPs) identified in herpesviruses and the *Molluscum contagiosum* virus (MSV) have also showed anti-necroptotic activity [98]. Together, these results indicate that necroptosis is a key defense mechanism against viral infection. 

In addition to viruses, necroptosis can occur through a variety of receptors, such as TNF-superfamily receptors, toll-like receptors, and interferon receptors. Moreover, toxins, genotoxic stress, and some anti-cancer drugs have been shown to initiate necroptosis [113,114,115,116,117].

Among them, TNFR-mediated necroptosis is the best-characterized necroptotic pathway (Figure 2), whose principal components are the protein kinases RIPK1 and RIPK3, and the MLKL.

#### 3.2.1. Initiation of Necroptosis

**Figure 3:**
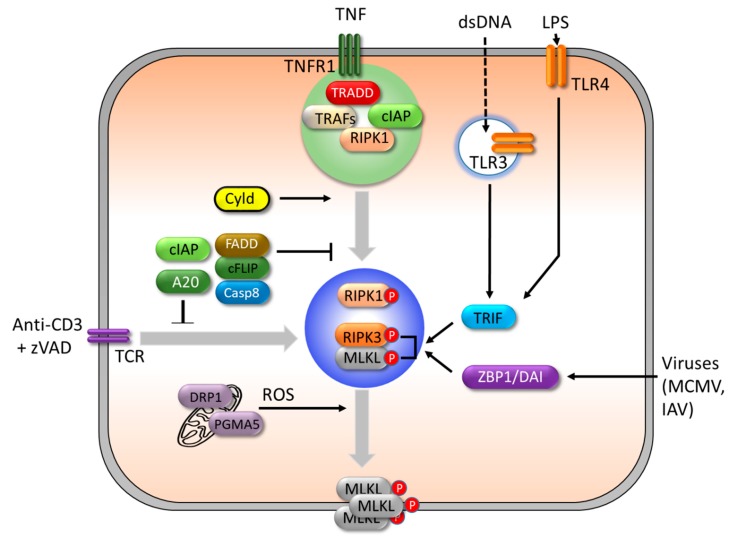
A simple depiction of the triggers and regulators of necroptosis. Upon TNF stimulation, cIAPs in the complex I inhibit necroptosis and execute cell survival signaling. Upon activation of TLRs by dsDNA, LPS, or viral infection, RIPK1-independent and RIPK3-mediated necroptosis is triggered by two RHIM-containing adaptor proteins, TRIF and ZBP1/DAI. T cell receptor triggering with caspase inhibitor (zVAD) also induces RIPK3-dependent necroptosis. The deubiquitination of RIPK1 by CYLD facilitates the formation of necrosome and promotes necroptosis. Another deubiquitinase, A20, also suppresses necroptosis through deubiquitinating RIPK1 or RIPK3. Phosphoglycerate mutase family member 5 (PGAM5) and Dynamin related protein 1 (Drp1) induce reactive oxygen species (ROS) production in the mitochondria and contribute to plasma membrane rupture.

RIPK1 and RIPK3 are known to be key regulators of TNF-induced necroptosis, and both of these kinases contain small protein domains called RIP homotypic interaction motifs (RHIMs) (Figure 2 and Figure 3). The ligation of TNF to its cognate receptor leads to the formation of large membrane receptor signaling complexes, where TRADD and RIPK1 are recruited first, followed by cIAPs and TRAF2/5. Then, RIPK1 is ubiquitinated by the cIAPs and TRAF2/5, which is crucial for initiating cell survival pathways, including NF-κB and MAPK activation. The NF-κB signaling pathway plays a key role in the protection of the deadly effect of TNF ligation [118,119]. However, if the activities of both cIAPs and caspase-8 are inhibited, RIPK1 forms a distinct protein complex with RIPK3 via RHIM–RHIM interactions, the protective effect of NF-κB is diminished, and the necroptotic death program is initiated [63,120,121,122]. Although we do not know precisely how RIPK1 activates RIPK3, the kinase activity of RIPK1 was demonstrated to be crucial [94,105]. It was reported that cells treated with necrostatin-1 (Nec-1), an RIPK1 kinase inhibitor, or the cells expressing catalytically inactive Ripk1 (D138N), are resistant to TNF- induced necroptosis [94,105]. Moreover, the mice expressing Ripk1 (D138N/D138N) were protected from TNF-induced death, and were unable to control the growth of the vaccinia virus [105]. In addition, the ablation of RIPK1 rescued the embryonic lethality of FADD- or caspase-8-deficient mice [123,124]. 

Although such an RIPK1–RIPK3 interaction is considered a hallmark of necroptosis, RIPK3 can trigger necroptosis in an RIPK1-independent manner [101,107,125]. In addition to RIPK1, other RHIM-containing proteins can trigger necroptosis through the interaction with RIPK3 in certain contexts [107,125]. For example, upon the stimulation of TLR3 and TLR4, RIPK3 is activated via interaction with the adaptor protein TIR domain-containing adaptor-inducing IFN-β (TRIF) and DNA sensor ZBP1/DAI [107,125]. In addition, ZBP1/DAI triggers RIPK3-mediated necroptosis through sensing viral RNA or cellular RNA in viral infected cells [126,127,128]. Furthermore, RIPK3 can interact with RHIM-containing proteins, such as RIPK1, TRIF, and ZBP1/DAI, to form an amyloid-like complex called necrosome, which facilitates RIPK3 phosphorylation [58,59,60]. The phosphorylated RIPK3 recruits MLKL and activates it by phosphorylation [129,130]. 

In earlier studies on necroptosis, phosphoglycerate mutase family member 5 (PGAM5) and dynamin related protein 1 (DRP1) were proposed to be downstream molecules of RIPK1/RIPK3 during death receptor- and oxidative stress-induced necrosis. PGAM5 was suggested to recruit the RIPK1/RIPK3 complex to the mitochondria to promote necroptosis through the dephosphorylation of the mitochondrial fission factor DRP1, which causes mitochondrial fragmentation for necroptosis [131].

However, many subsequent studies have challenged the role of the PGAM5/DRP1 axis on necroptosis, as many studies have shown that the silencing of PGAM5 and DRP1 did not influence the necroptotic processes [132,133,134]. 

#### 3.2.2. Execution of Necroptosis 

As mentioned above, RIPK3 with RHIM-containing molecules is an initiator of necroptosis, whereas MLKL works as the executioner of necroptosis. MLKL was identified as a target molecule of necrosulfonamide (NSA), a chemical necroptosis inhibitor, and it is composed of an N-terminal coiled-coil region and a C-terminal kinase-like domain [131]. 

MLKL is recruited to the necrosome via interaction with RIPK3 and is phosphorylated by activated RIPK3 in its kinase-like domain [99,135]. The phosphorylated MLKL undergoes a conformational change and oligomerization that enables it to bind phosphatidylinositol lipids and cardiolipin [130,136]. The oligomerized MLKL then translocates to the plasma or intracellular membranes, and executes necroptosis by the disruption of the membrane integrity [130,136]. However, a recent study proposed that the active MLKL translocates to the membrane, where it oligomerizes and acts as an effector for necroptosis [137]. Further work is required in order to clarify the exact pathway for MLKL translocation.

Several groups have sought to determine a mechanism of MLKL function in necroptosis execution. Some have suggested that activated MLKL binds to the phospholipid and makes pores in the plasma membrane [130,136,138], while others have proposed that the localization of MLKL to the plasma membrane induces an influx of ions, either through association with ion channels or via pore formation [135,139,140]. Despite these attempts, however, our knowledge of how MLKL executes cell death remains limited.

#### 3.2.3. Regulation of Necroptosis. 

Necroptosis is tightly controlled by several regulators, including caspase-8, cFLIP, FADD, and deubiquitinases (Figure 3).

Although caspase-8 has long been considered to reside in the domain of apoptosis, targeted gene disruption in mice has revealed that caspase-8 also has a pro-survival function [141]. The genetic ablation of caspase-8 leads to embryonic lethality around E10.5 [141]. Intriguingly, the knockout of cFLIP also results in embryonic lethality at the same age [142]. Moreover, both types of mice can be rescued from death by the knockout of RIPK3, a master molecule of necroptosis [63], which points to an inhibitory role of caspase-8 and cFLIP in necroptosis. 

As discussed above, c-FLIP counteracts the function of caspase-8 in the apoptotic cell death pathway [143], but works collaboratively for the inhibition of necroptosis [64]. It has been reported that caspase-8/cFLIP association and the catalytic function of caspase-8 are necessary to inhibit TNF-induced necroptosis [64,144], and several molecules, including RIPK1, RIPK3, and CYLD, have been proposed as substrates of capase-8 [145,146]. 

As described above, deubiquitinated RIPK1 can interact with RIPK3, FADD, cFLIP, and caspase-8 to form ripoptosome, and it has been known that RIPK1 and RIPK3 contain caspase-8 cleavage sites [145,147]. Therefore, it has been suggested that the recruited caspase-8, in coordination with cFLIP, cleaves RIPK1 or RIPK3 [78,148]. 

The deubiquitinase, CYLD, has been suggested to be another target of caspase-8 to generate a survival signal. CYLD has been demonstrated to promote necroptosis [89], by facilitating RIPK1-translocation from the receptor-bound complex to the cytosolic RIPK1–RIPK3 complex [149]. Moreover, the expression of CYLD mutated at the caspase-8 cleavage site Asp 215 has been shown to switch cells from survival to necrotic cell death in response to TNF [146]. To our knowledge, no direct evidence has been presented of where or how exactly CYLD targets its substrates and regulates necroptosis signaling, and this still needs to be clarified [146]. 

Unlike CYLD, A20 acts as a negative regulator of necroptosis [150]. A20-deficient T cells have shown more susceptibility to death upon T cell receptor stimulation by anti-CD3 antibodies in the presence of a pan-caspase inhibitor, zVAD, while RIPK3 deficiency significantly protects activated T cells from death [150]. Moreover, A20-deficient embryonic fibroblasts (MEFs) exhibit a ubiquitination of RIPK3 at K5 and an exaggerated formation of RIPK1–RIPK3 complexes upon stimulation with TNF in the presence of cycloheximide and zVAD [150]. As the authors determined that both RIPK3 ubiquitination and the formation of the RIPK1–RIPK3 complex require the catalytic cysteine of A20′s deubiquitinating motif, A20 might suppress necroptosis through deubiquitinating RIPK1 or RIPK3 [150].

## 4. Cell Death Regulators Associated with the Inflammasome.

Several molecules have been shown to be involved in inflammasome activation and IL-1β processing, in addition to their roles in cell death.

### 4.1. Caspase-8 and cFLIP

**Figure 4:**
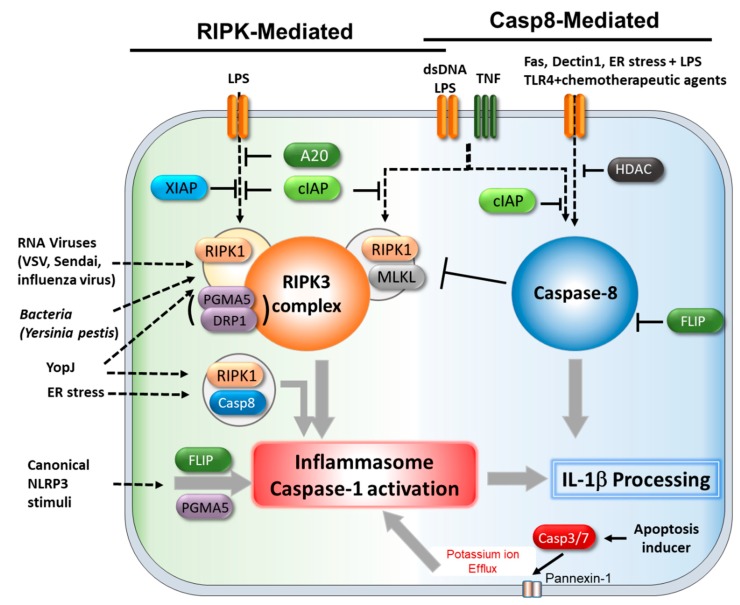
Roles of necroptosis molecules in inflammasome activation and IL-1β processing. Apoptosis- or necroptosis-related molecules contribute to inflammasome activation and IL-1β processing through two distinctive pathways. One is through the protein kinase RIPK-complex, the other one is through caspase-8. Caspase-8 can directly cleaves pro-IL-1β upon stimulation with stimuli such as FasL, Dectin-1, and LPS combined with chemotherapeutic drugs, as well as the inhibition of HDAC. The caspase-8 function in these processes could be inhibited by c-FLIP. Caspase-8 can also promote the activation of inflammasome or caspase-1 in response to YopJ and endoplasmic reticulum (ER) stress. The disruption of some genes, including A20, cIAPs, and XIAP, induces RIPK3-complex-mediated inflammasome activation upon stimulation with a TLR-agonist and TNF. Pathogenic infection, such as RNA viruses and bacteria, induce inflammasome activation through the RIPK1–RIPK3 complex. Depending on the stimuli, the RIPK1–RIPK3 complex promotes inflammasome activation with the help of DRP1 and PGAM5. PGAM5 and c-FLIP contribute to canonical NLRP3 inflammasome activation induced by LPS and ATP or monosodium urate (MSU). The disruption of caspase-8 leads to MLKL–RIPK3-mediated inflammasome activation. A caspase-3/-7-mediated pannexin-1activation during apoptosis promotes NLRP3 inflammasome via potassium efflux.

Caspase-8 has been proposed to have a role in some inflammasome activation in the IL-1β secretory system, and it appears to have dual functions, both as a positive regulator and as a negative regulator [151,152,153,154,155,156,157,158] (Figure 4). 

Caspase-8 has been shown to contribute to IL-1β processing in two different ways, either by the direct cleavage of IL-1β or by the activation of NLRP3-caspase-1.

To date, several studies have implicated caspase-8 in the secretion and processing of bioactive IL-1β, depending on the cell type and stimuli [151,152]. For example, when the protein synthesis was inhibited in caspase-1-deficient cells, poly(I:C)- and LPS-induced pro-IL-1β processing was mediated by caspase-8 [151]. Moreover, recombinant caspase-8 was able to cleave pro-IL-1β at the same site as caspase-1 [151], suggesting that caspase-8 can be a substitute for caspase-1 in some conditions. Under the condition of cIAP inhibition, the cleavage of IL-1β by TLR stimulation can be mediated by caspase-8, as well as by NLRP3- inflammasome [152]. Moreover, the inhibition of histone deacetylases (HDAC), endoplasmic reticulum stress, and treatment with chemotherapeutic agents were shown to promote TLR-induced IL-1β processing and secretion in macrophages or dendritic cells in a caspase-8 dependent pathway [153,154,159]. Caspase-8 mediated IL-1β processing also occurs upon stimulation with FasL or Dectin-1 in certain contexts [155,160]. 

In addition to the direct cleavage of IL-1β, caspase-8 also functions to activate inflammasome. It has been reported that caspase-8 is recruited to activate NLRP3 inflammasomes to drive IL-1β processing in murine bone marrow-derived dendritic cells (BMDCs), independently of caspase-1 and -11 [161]. Moreover, caspase-8 was shown to be required for TLR3-induced NLRP3 priming [162]. Another study revealed that caspase-8, together with FADD, drives potent NLRP3 inflammasome activation at both the transcriptional- and post-translational level [163].

Caspase-8 may also have a role in other types of inflammasome activation, other than NLRP3 [164,165]. Upon *Salmonella* infection, caspase-8 has been found to associate with the NLRC4 inflammasome complex and thus contribute to *Salmonella*-induced IL-1β processing in macrophages [164]. Moreover, another study provided evidence that caspase-8 is an integral part of inflammasome, recruited through its binding to the pyrin domain of ASC [165]. 

It seems that caspase-8 also promotes NLRP3 inflammasome activation in an indirect way. Recent studies have proposed that caspase-8 activates GSDMD or the channel-forming glycoprotein pannexin-1 by cleaving them during extrinsic apoptosis, which promotes the formation of plasma membrane pores and elevates potassium efflux, resulting in activation of the NLRP3 inflammasome [166,167].

Together, caspase-8 might contribute to IL-1β processing through the direct cleavage of pro-IL-1β, by associating with the inflammasome, or by activating pore-forming proteins. 

However, in some cases, caspase-8 also has an inhibitory function on RIPK3-MLKL-mediated inflammasome activation and IL-1β processing [156,157]. The ablation of caspase-8 in BMDCs is sufficient to cause NLRP3 activation and IL-1β processing upon TLR stimulation in an RIP3-MLKL dependent manner [156]. In addition, another study has also shown that in the absence of both cIAPs, caspase-8 loss promotes TLR-induced NLRP3 activation in macrophages in an RIPK3-MLKL-dependent manner [157,158]. 

The impact of c-FLIP on inflammasome activation was first acknowledged in the studies of c- FLIP^+/−^ macrophages, where it was found to have contrasting effects on IL-1β processing mediated by canonical inflammasome and caspase-8 [168]. The c-FLIP downregulation decreased NLRP3- and AIM2-mediated IL-1β production in the TLR-primed macrophages, while having no effect on the production of TNF-α, indicating that c-FLIP might be a positive regulator of inflammasome activation [168]. Mechanistically, c-FLIP has been reported to interact with NLRP3 inflammasome, facilitating its optimal assembly and mitochondrial localization [168]. However, the same study showed that the hemizygotic deficiency of c-FLIP increases the FasL- and Dectin-1 induced IL-1β production, which is mediated by caspase-8 [168]. Therefore, the functions of c-FLIP in IL-1β processing is likely dependent on stimuli or cellular context. 

### 4.2. A20

A20 has been shown to be involved in the regulation of inflammasome activity as well [169]. The deficiency of A20 in macrophages leads to the increased formation of the RIPK1–RIPK3 complex and spontaneous RIPK3-deriven NLRP3-inflammasome activation in response to LPS alone [169]. Moreover, NLRP3 was hyperactivated in response to canonical stimuli, including ATP, nigericin, and silica, in A20 deficient macrophages [170]. 

Mechanistically, A20 has been suggested to restrict inflammasome activity at both the transcriptional and post-translational level. A20 was recruited for the NLRP3–ASC inflammasome and to prevent its spontaneous activation through the restriction of pro-IL1β-ubiquitination, which supports its processing [169]. Transcriptionally, A20 was shown to limit the basal- and LPS-induced upregulation of the NLRP3 expression and the level of pro-IL-1β and pro-IL-18 [170]. 

### 4.3. RIPK1/3

Several studies have documented that RIPK3 mediates the activation of NLRP3 inflammasome through the formation of complexes with different molecules, depending on the stimuli and cellular context (Figure 4). 

The targeted disruption of several genes has been reported to sensitize cells to inflammasome activators. It was further demonstrated that the activation of RIPK3 signaling is sufficient for the induction of IL-1β maturation and secretion. The deficiency of the X-linked inhibitor of the apoptosis protein (XIAP) leads to RIPK3-mediated inflammasome activation in BMDCs, and potentiates the induction of IL-1β in mice in response to LPS [171]. Moreover, a similar result was observed in LPS- or TNF- primed XIAP-deficient BMDMs, where RIPK3-dependent NLRP3 inflammasome activation occurs. [157]. 

In line with these findings, when cIAPs are inhibited, the LPS stimulation of macrophages leads to the cleavage of IL-1β by both the NLRP3-caspase-1 inflammasome and caspase-8. Moreover, both caspase-1- and caspase-8 activation requires the kinase activity of RIPK3 [152]. In addition, caspase-8-deficient BMDCs also shows NLRP3-inflammasome activation in response to LPS alone, and inflammasome activation is abolished by the ablation of RIPK3 incaspase-8 deficient BMDCs [156]. 

In most cases, RIPK3 functions as a complex with RIPK1 to activate inflammasome. *Yersinia pestis* infection triggers RIPK1–RIPK3 complex-dependent caspase-1 activation [172]. Similarly, many other pathogenic infections and the *Yersinia* outer protein J (Yop J) trigger RIPK-mediated inflammasome activation [134,172,173]. Infection by some RNA viruses, including vesicular stomatitis virus (VSV), Sendai virus, and influenza virus, induces NLRP3 inflammasome activation, and the silencing of RIPK1 and RIPK3 leads to a severe reduction in inflammasome activation [134]. Mechanistically, it has been suggested that the viral infection induces RIPK1–RIPK3 complex-mediated mitochondrial damage through DRP1, followed by inflammasome activation [134].

In another study, RIPK3 was found to promote IL-1β secretion through interaction with caspase-8. The authors observed that mere stimulation with LPS can prompt the secretion of substantial amounts of mature IL-1β in the absence of a second stimulation in BMDCs [174]. Mechanistically, RIPK3 functions as a positive regulator of IL-1β secretion by enhancing the assembly of the alternative caspase-8 activating complex, composed of RIPK1 and FADD [174].

Some stimuli can induce RIPK1-dependent inflammasome activation without the assistance of RIPK3. For example, endoplasmic reticulum (ER) stress by the ER stress-inducing drug thapsigargin is shown to induce inflammasome activation in BMDM, and such an activation is severely reduced by RIPK1 silencing but not by RIPK3 silencing [175], indicating that ER stress-induced inflammasome activation is mediated by RIPK1 without contribution of the RIPK3. It was also reported that YopJ induces caspase-1 activation and IL-1β processing through the formation of a RIPK1/FADD/caspase-8 complex that does not contain RIPK3 [172,173].

In human monocytes, the LPS application has been known to induce IL-1β secretion via the so-called alternative inflammasome pathway, which bypasses classical inflammasome processes, such as K^+^ efflux and pyroptosis [57], however, RIPK1 was required as an upstream signaling molecule for the inflammasome activation in this model [57].

Interestingly, compared to the canonical inflammasome activation, which requires both the priming and the activation signals, most of the known instances of RIPK complex-mediated inflammasome and caspase-1 activation occur in the absence of an activation signal. 

### 4.4. MLKL

To date, several studies have documented the impact of MLKL on inflammasome activation, and most examples of MKLK-mediated inflammasome activation occur in the absence of caspase-8 activity [156,157,176,177]. Caspase-8 deficiency in BMDCs was shown to facilitate NLRP3 inflammasome activation upon LPS stimulation, which was prevented by the silencing of MLKL [156,176]. 

Other groups have shown that the MLKL activation itself is sufficient to trigger a potassium efflux and the assembly of the NLRP3 inflammasome, leading to caspase-1 dependent processing of IL-1β [167,176]. Moreover, MLKL activation-induced cell membrane disruption allows for the release of IL-1β, independently of the pyroptotic effector gasdermin D [167,176]. 

Moreover, when cIAPs are blocked, LPS triggers RIPK3-caspase-8 to promote NLRP3-caspase-1 activation, independent of MLKL. In contrast, when both IAPs and caspase-8 activities are blocked, RIPK3 kinase activity and MLKL are required for TLR-induced NLRP3 activation [157], which indicates that MLKL-mediated inflammasome activation is restricted by caspase-8. 

MLKL may also contribute to inflammasome activation through the transcriptional regulation of NLRP3 and pro-IL-1β expression in a cellular context-dependent fashion [177]. MLKL/FADD double deficient BMDMs have shown impaired NLRP3 inflammasome activation upon stimulation with LPS and ATP, because of the lack of NLRP3 expression due to impaired NF-kB activation [177]. However, in both studies, the inflammasome activation by classical stimuli (TLR and ATP) in MLKL-deficient BMDMs is comparable to that of wild-type BMDMs [157,177], which implies that the impact of MLKL on inflammasome is highly dependent on the cellular context.

### 4.5. PGAM5/DRP1

Although the roles of PGAM5 and DRP1 in the necroptosis have been challenged [132,178], several studies have suggested their role in inflammasome activation [156,178]. PGAM5 was first implicated in NLRP3 inflammasome activation and IL-1β secretion in caspase 8-deficient BMDCs [156]. Moreover, the deficiency of PGAM5 results in a significantly reduced IL-1β secretion from the LPS-primed BMDMs, induced by ATP, nigericin, and poly (dA-dT), indicating its critical role in NLRP3 or AIM2 inflammasome activation [178].

DRP1 is activated by the RIPK1–RIPK3 complex, formed in cells infected with RNA viruses, and translocates to the mitochondria to promote NLRP3 inflammasome activation, which establishes the role of the RIPK1–RIPK3–DRP1 pathway in NLRP3 inflammasome activation [134]. In line with this finding, swine influenza virus-infection leads to NLRP3 inflammasome activation and IL-1β secretion in porcine alveolar macrophages in an RIPK1/DRP1 complex-dependent manner, which indicates the critical role of the RIPK1/DRP1 signaling axis in modulating porcine NLRP3 inflammasome activation [179]. The RIPK1/RIPK3/DRP1 pathway has also been proposed to contribute to the activation of NLRP3 inflammasome in the subarachnoid hemorrhage induction animal model [180]. More recently, it was suggested that DRP1, as a downstream molecule of RIPK1, mediates ER-stress-induced inflammasome activation in murine macrophages [175]. 

Although the exact mechanism underlying DRP1-mediated inflammasome activation needs to be clarified, the reactive oxygen species (ROS) generation during mitochondrial fission was suggested to be the key mediator [134,175,180,181]. However, PGAM5 deficiency does not significantly affect the ROS generation induced by the NLRP3 inflammasome agonist, which indicates that PGAM5-mediated inflammasome activation might be carried out by other mechanisms [178]. 

## 5. Conclusions

Recent studies have demonstrated the existence of cross-talk among many signaling pathways that have been thought of, for a long time, as being biochemically separate from each other. In line with this notion, many studies have indicated interrelationships between cell death and inflammasome signaling. The critical regulators of cell death machinery, such as cIAPs, RIPK1/RIPK3, caspase-8/c-FLIP, and MLKL, can directly and indirectly modulate inflammasome activation and IL-1β processing in response to PAMPs and DAMPs. Moreover, current evidence suggests that their function in such pro-inflammatory processes is independent of cell death. However, the data that documents the impact of apoptotic and necroptotic signaling molecules on inflammasome are varied, and are highly dependent on the cell type and cellular context. 

Therefore, we are still some way from having a comprehensive picture of how exactly these molecules contribute to inflammasome activation under different conditions and why these cell death pathways have evolved to function in inflammasome activation. Consequently, it will also be of interest to investigate under what specific conditions apoptotic-, necroptosis-, and inflammasome-mediated IL-1β secretion is required for host defense.
